# Parapatric genetic divergence among deep evolutionary lineages in the Mediterranean green crab, *Carcinus aestuarii* (Brachyura, Portunoidea, Carcinidae), accounts for a sharp phylogeographic break in the Eastern Mediterranean

**DOI:** 10.1186/s12862-018-1167-4

**Published:** 2018-04-11

**Authors:** Temim Deli, Evrim Kalkan, Selahattin Ünsal Karhan, Sonya Uzunova, Alireza Keikhosravi, Raşit Bilgin, Christoph D. Schubart

**Affiliations:** 1 Laboratory of Genetics, Biodiversity and Enhancement of Bioresources (LR11ES41), University of Monastir, Higher Institute of Biotechnology of Monastir, Av. Tahar Hadded, B.P. 74, 5000 Monastir, Tunisia; 2 Middle East Technical University, Institute of Marine Sciences, P.O.Box 28, 33731 Erdemli-Mersin, Turkey; 3 19 Mayis Mah., Gürsoylu Sok., Zambak Apt. No: 39/1, Kadiköy, 34736 Istanbul, Turkey; 4 Institute of Fish Resources – Varna, Agricultural Academy, Primorski, 4 blvd, 9000 Varna, Bulgaria; 5 Department of Biology, Hakim Sabzevari University, Sabzevar, Iran; 6 Institute of Environmental Sciences, Boğaziçi University, Bebek, 34342 Istanbul, Turkey; 7 Zoology & Evolutionary Biology, Universität Regensburg, D-93040 Regensburg, Germany

**Keywords:** Crustacea, Population genetics, Biogeographic boundaries, Evolutionary history, Mitochondrial DNA, Mediterranean Sea

## Abstract

**Background:**

Recently, population genetic studies of Mediterranean marine species highlighted patterns of genetic divergence and phylogeographic breaks, due to the interplay between impacts of Pleistocene climate shifts and contemporary hydrographical barriers. These factors markedly shaped the distribution of marine organisms and their genetic makeup. The present study is part of an ongoing effort to understand the phylogeography and evolutionary history of the highly dispersive Mediterranean green crab, *Carcinus aestuarii* (Nardo, 1847), across the Mediterranean Sea. Recently, marked divergence between two highly separated haplogroups (genetic types I and II) of *C. aestuarii* was discerned across the Siculo-Tunisian Strait, suggesting an Early Pleistocene vicariant event. In order to better identify phylogeographic patterns in this species, a total of 263 individuals from 22 Mediterranean locations were analysed by comparing a 587 basepair region of the mitochondrial gene Cox1 (cytochrome oxidase subunit 1). The examined dataset is composed of both newly generated sequences (76) and previously investigated ones (187).

**Results:**

Our results unveiled the occurrence of a highly divergent haplogroup (genetic type III) in the most north-eastern part of the Mediterranean Sea. Divergence between the most distinct type III and the common ancestor of both types I and II corresponds to the Early Pleistocene and coincides with the historical episode of separation between types I and II. Our results also revealed strong genetic divergence among adjacent regions (separating the Aegean and Marmara seas from the remaining distribution zone) and confirmed a sharp phylogeographic break across the Eastern Mediterranean. The recorded parapatric genetic divergence, with the potential existence of a contact zone between both groups in the Ionian Sea and notable differences in the demographic history, suggest the likely impact of paleoclimatic events, as well as past and contemporary oceanographic processes, in shaping genetic variability of this species.

**Conclusions:**

Our findings not only provide further evidence for the complex evolutionary history of the green crab in the Mediterranean Sea, but also stress the importance of investigating peripheral areas in the species’ distribution zone in order to fully understand the distribution of genetic diversity and unravel hidden genetic units and local patterns of endemism.

**Electronic supplementary material:**

The online version of this article (10.1186/s12862-018-1167-4) contains supplementary material, which is available to authorized users.

## Background

In the marine environment, genetic variability and population genetic structure of a species are shaped by both contemporary and historical marine barriers, such as narrow and shallow water passages between landmasses [1], salinity gradients [2], different types of currents [3], as well as palaeoecological history [4, 5]. In particular, historical processes have left marked imprints on the genetic structure of extant marine populations. For example, the extent of genetic divergence between genomes varies among marine species and can be used to estimate the time of their separation. Marine populations inhabiting specific regions (i.e., those confined to historical refugial zones) might have harboured persisting genetic lineages through time and therefore accumulated genetic differences, leading to their possible speciation through several climatic cycles (i.e., the Quaternary glaciations periods) [6].

Palaeoclimatic and palaeogeographical evolution of the Mediterranean, primed by sea-level low stands during glacial periods of the Pleistocene and the consequent shift in abiotic (i.e., temperature and salinity) and biotic factors (i.e., productivity), prompted population isolation and divergence, following local events of extinction and recolonization. This in turn enhanced the formation of intraspecific genetic units within various marine taxa, due to historical or contemporary hydrographic barriers to gene flow [4, 5]. The best known oceanographic discontinuities in the Mediterranean are: (1) the Almería-Oran Front as the main genetic breakpoint between the Atlantic Ocean and the Mediterranean Sea [4, 7], (2) the transition between the Western and the Eastern Mediterranean, due to unidirectional water flow at the Siculo-Tunisian Strait [5], and (3) the hydrographic isolation of the Aegean, Ionian and Adriatic seas [5]. In addition to these oceanographic barriers, the geological history of the Eastern Atlantic and Mediterranean Sea, including the breaking up of the Tethys Sea, the Messinian Salinity Crisis [8], and the Pleistocene glaciations [9], might have left marked footprints on the genetic structure of species and made the Mediterranean region a notably dynamic hotspot of diversity [10–12]. For example, intensified modifications to the coastline (sea level regressions) during repeated Pleistocene glaciations could have limited the biotic exchange across physical barriers, such as the Gibraltar Strait [13] and the Siculo-Tunisian Strait [14], and strongly influenced the formation of distinct phylogenetic lineages within Mediterranean marine species.

In this context, the phylogeography of Mediterranean species underwent a common set of processes resulting from fragmentation within glacial refugia, range expansions via postglacial colonisation, and secondary contact zones among historically divergent lineages [4, 12, 15–18].

The Mediterranean green crab, *Carcinus aestuarii* (Nardo, 1847) (Brachyura, Portunoidea, Carcinidae), represents a good model to test the impact of current and historical marine barriers on population subdivision and genetic structuring across various marine biogeographic boundaries in the Mediterranean Sea. This species is a very common inhabitant of estuaries and lagoons of the Mediterranean Sea and has also been reported from the southern Black Sea [19, 20]. It inhabits various types of environments, ranging from sheltered and often brackish habitats (including subtidal and intertidal mud, as well as sand flats at open coastal sites or lagoons and estuaries) to saltmarshes, and seagrass beds (authors’ personal observations). It is a voracious omnivore and aggressive competitor. Furthermore, it has a wide tolerance toward variations of salinity, temperature, and dissolved oxygen. Hence, it has a high ability to adapt to a wide variety of habitats [19, 21]. The Mediterranean green crab also exhibits high fecundity and a relatively long dispersal stages, with a larval planktonic phase of approximately six weeks [22, 23].

Owing to maritime commerce and ballast transport, several reports have pointed out to the accidental introduction of *C. aestuarii* specimens into several regions outside their native range, such as to the Canary Islands [24], Tokyo Bay, Japan [25] and South Africa [22, 26].

Among the above mentioned eco-biological characteristics of *C. aestuarii*, the particular ecological specialization of this species to estuarine and brackish-water habitats in the Mediterranean could be considered as important factor susceptible of generating significant phylogeographic patterns, owing to limited connectivity among estuarine populations. Restricted gene flow is highly expected in estuarine and brackish-water taxa owing to larvae retention within their natal estuary (e.g. [27–29]). In addition to this mechanism, geographically extended distances separating estuaries (e.g. [29]), the differential in physical characteristics of estuarine and their adjacent coastal environments [30], as well as the potential different physiological challenges between estuarine and coastal waters [31], could also impede inter-estuarine larval dispersal. Numerous investigations have shown that genetic connectivity among populations of estuarine taxa is more restricted than that occurring in taxa inhabiting the open coast [27, 32, 33].

Recent population genetic surveys on *C. aestuarii*, across native and invaded regions [22, 23, 34, 35], unveiled extensive genetic variability and marked population differentiation associated with the main oceanographic discontinuities that characterize these areas. In particular, Marino et al. [23] and Ragionieri and Schubart [35] found significant genetic differentiation among populations from western and eastern Mediterranean coastlines in Europe. This noticeable pattern of genetic structure has been later confirmed across the central African Mediterranean coast, when Deli et al. [36] revealed a sharp haplotypic discontinuity among eastern and western sites in Tunisia. Both retrieved genetic groups were found to be genetically and morphologically differentiated across the Siculo-Tunisian Strait [36, 37]. A more recent investigation by Deli et al. [12], detailing phylogeography and population genetic structure of the Mediterranean green crab across the Siculo-Tunisian Strait, showed concordant patterns of mitochondrial and nuclear divergence among western and eastern Mediterranean populations from northern Africa. The study also revealed a marked divergence between two highly separated haplogroups, suggesting an Early Pleistocene vicariant event in the genetic differentiation of this highly dispersive decapod species [12].

These insights, inferred so far from population genetic surveys of *C. aestuarii* throughout the western and central Mediterranean coasts [12, 23, 34–36], trigger the necessity of detailed phylogeographic and population genetic analyses of this species across the poorly investigated Eastern Mediterranean Basin. Sea-level Pleistocene oscillations potentially caused the isolation or partial isolation of the Black Sea, Aegean Sea and Eastern Mediterranean Basin [38]. These historical isolation processes have been maintained by the impact of the contemporary hydrographic isolation of the Adriatic, Ionian and Aegean seas [4, 5]. In addition to these historical and contemporary hydrographic isolation patterns, different selective forces related to the environmental conditions of the Ionian and Aegean seas could account for the phylogeographical break observed in the Eastern Mediterranean. Previous population genetic studies on many vertebrate and invertebrate species confirmed this tendency and identified a major genetic break related to the hydrographic isolation of the Aegean Sea [5, 16, 39–45].

In light of these considerations, it will be interesting to re-examine population genetic structure in a highly dispersive decapod species, like *Carcinus aestuarii*, across these potential barriers to gene flow. This may contribute to a better identification of the phylogeographic patterns in this species and unveil other potential evolutionary lineages within *C. aestuarii* across the poorly investigated easternmost part of its distribution range. It may also allow depicting the historical events that might have shaped the genetic structure of the Mediterranean green crab. In order to achieve these goals, new mitochondrial Cox1 (cytochrome oxidase subunit 1) sequences were obtained from 76 specimens collected from seven sites across the Eastern Mediterranean Basin, stretching across the Greek and Turkish coasts. This newly generated dataset of sequences was merged with previously examined data by Ragionieri and Schubart [35] and Deli et al. [12] for phylogeographic re-analyses.

## Methods

### Sampling strategy and genomic DNA extraction

**Fig. 1:**
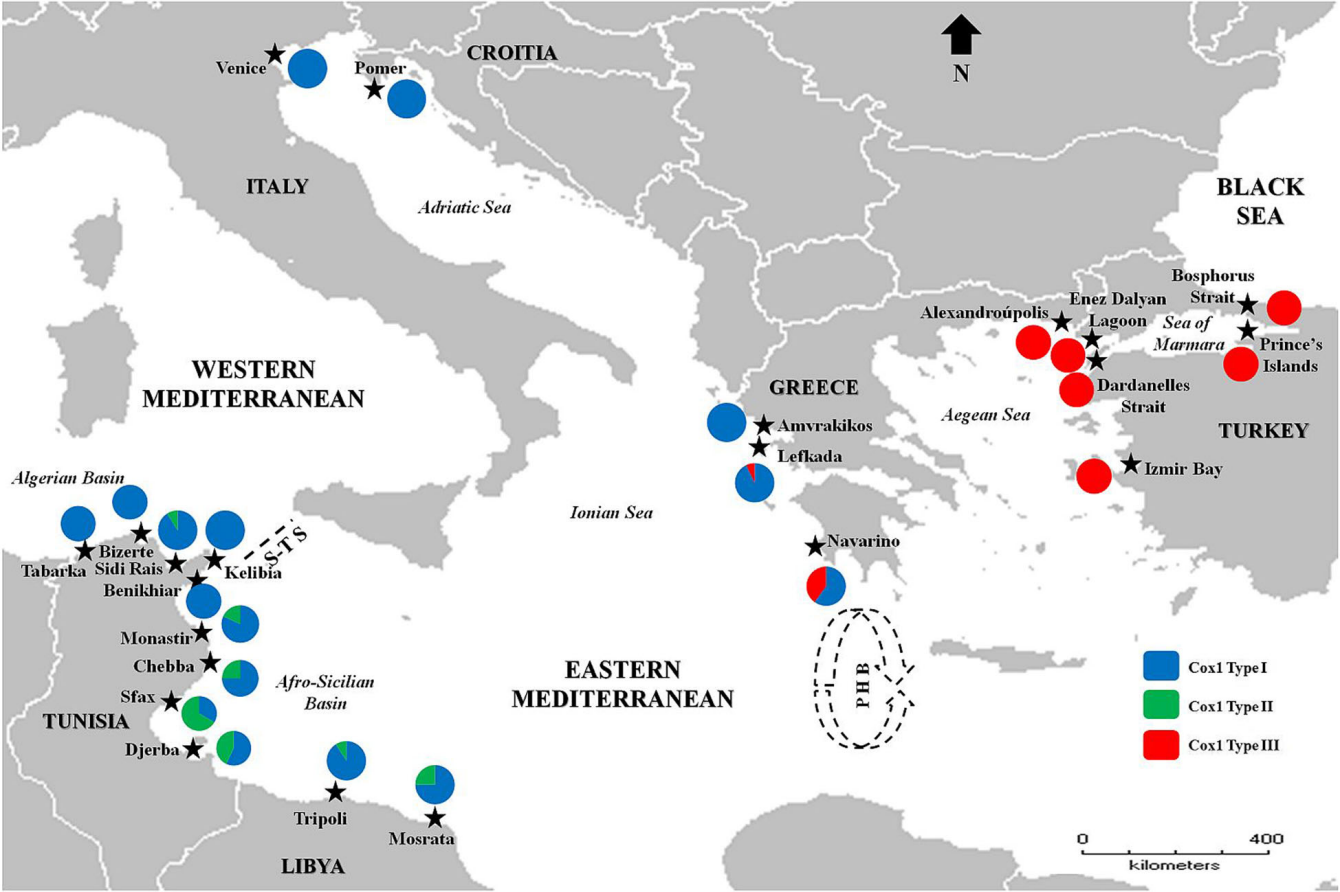
Sampling locations of the green crab *Carcinus aestuarii* across the Mediterranean Sea. Distribution patterns and proportions of Cox1 types (I, II, and III) along the examined locations are shown in couloured circles. S-T S: Siculo-Tunisian Strait; PHB: Peloponnese Hydrographic Break (represented by the quasi-circular anti-cyclonic feature southwest of Peloponnese). The base map was constructed with the software DIVA-GIS 7.5.0 (http://www.diva-gis.org)

A total of 263 samples of *Carcinus aestuarii* were included in the present phylogeographic study, of which 76 were newly analyzed, and the remaining 187 had been previously investigated [12, 35] (Table 1 and Fig. 1). Newly examined specimens of *C. aestuarii* were collected from seven locations stretching across the Greek (Lefkada and Alexandroupolis) and Turkish coasts (Izmir Bay and Enez Dalyan Lagoon in the Aegean Sea; Dardanelles Strait, Prince’s Islands and Bosphorus Strait in the Sea of Marmara) (Fig. 1). Previously examined data by Ragionieri and Schubart [35] include the populations of Pomer (Croatia), Amvrakikos, and Navarino (both Greece) corresponding to the Adriatic and Ionian seas respectively; while those retrieved from Deli et al. [12] comprise all surveyed populations from North Africa and Venice Lagoon. In the present study, we included all previously investigated eastern Mediterranean populations in order to optimize the population genetic structure analysis across the poorly surveyed Eastern Mediterranean Basin, whereas from the available western Mediterranean locations we only included those from Deli et al. [12]. This strategy was adopted, as the primary aim of this study is the examination of the phylogeography and evolutionary history of eastern Mediterranean populations of *C. aestuarii*. Western Mediterranean locations are included as reference populations to re-analyze population genetic structure and test for genetic subdivision across the Siculo-Tunisian Strait. Those from the North-African coast were included in this study, because they allowed Deli et al. [12] to confirm and delineate the geographic break across the Siculo-Tunisan Strait based on the concordant patterns of mitochondrial and nuclear phylogeographic structure. This choice has been validated after confirming that incorporation of additional western Mediterranean sequences from Ragionieri and Schubart [35] did not affect the outcome of phylogeographic analyses and population structure. From each crab, muscle tissue was dissected from a removed pereiopod (after releasing the animal back into its original environment) and stored in absolute ethanol at − 20 °C until genetic analysis. Total genomic DNA was isolated from muscle tissue using the Wizard® genomic DNA purification kit (Promega), the Puregene kit (Gentra Systems: Minneapolis, MN55447, USA) or the Roche High Pure PCR Template Preparation Kit (Indianapolis, USA) following the instructions of the suppliers.

**Table 1:** Sampling information on the green crab *Carcinus aestuarii* including collection sites, countries, Mediterranean basins, regions, geographic coordinates, and number of examined specimens (N) per each location. Genetic diversity measures (including number of haplotype (*N*h), number of polymorphic sites (*N*ps), haplotype (*h*) and nucleotide (*π*) diversities, and mean number of nucleotide differences (K)) and historical demographic results (inferred from: Tajima’s *D* test (*D*), Fu’s *F*_*S*_ test (*F*_*S*_), Ramos-Onsins and Rozas’s *R*_2_ test (*R*_2_), and mismatch distribution raggedness index (*rg*)) were also provided for each investigated location and the total dataset

Collection site	Country	Mediterranean Basin	Region	Geographic coordinates	N	*N*h	*N*ps	*h*	*π*	K	*D*	*F*_*S*_	*R*_*2*_	*rg*
Tabarka^b^	Tunisia	Western Mediterranean	Algerian Basin	36°57′N 08°45′E	10	5	6	0.756 ± 0.130	0.0023 ± 0.0006	1.355	**−1.492**	−1.507	0.137	0.042
Bizerte^b^	Tunisia	Western Mediterranean	Algerian Basin	37°16′N 09°52′E	15	8	10	0.867 ± 0.067	0.0030 ± 0.0005	1.771	**−1.613**	**−3.541**	**0.083**	0.064
Sidi Rais^b^	Tunisia	Western Mediterranean	Algerian Basin	36°46′N 10°32′E	11	5	21	0.709 ± 0.137	0.0072 ± 0.0045	4.236	**−1.859**	1.435	0.258	0.094
Kelibia^b^	Tunisia	Western Mediterranean	Afro-Sicilian Basin	36°51′N 11°05′E	10	5	6	0.667 ± 0.163	0.0023 ± 0.0008	1.355	**−1.492**	−1.507	0.137	0.037
Benikhiar^b^	Tunisia	Western Mediterranean	Afro-Sicilian Basin	36°28′N 10°46′E	12	2	1	0.167 ± 0.134	0.0002 ± 0.0002	0.166	−1.140	−0.475	0.276	0.472
Monastir^b^	Tunisia	Eastern Mediterranean	Afro-Sicilian Basin	36°10′N 10°49′E	11	9	28	0.945 ± 0.066	0.0138 ± 0.0051	8.127	−0.692	−1.391	0.120	0.054
Chebba^b^	Tunisia	Eastern Mediterranean	Afro-Sicilian Basin	35°14′N 11°07′E	12	11	31	0.985 ± 0.040	0.0160 ± 0.0039	9.424	−0.369	−3.052	0.118	0.051
Sfax^b^	Tunisia	Eastern Mediterranean	Afro-Sicilian Basin	34°44′N 10°45′E	15	12	27	0.943 ± 0.054	0.0166 ± 0.0025	9.752	0.729	−2.077	0.160	0.026
Djerba^b^	Tunisia	Eastern Mediterranean	Afro-Sicilian Basin	33°52′N 10°51′E	14	9	29	0.912 ± 0.059	0.0186 ± 0.0022	10.967	0.867	0.813	0.166	0.044
Tripoli^b^	Libya	Eastern Mediterranean	Afro-Sicilian Basin	32°54′N 13°11′E	11	6	23	0.891 ± 0.063	0.0089 ± 0.0043	5.254	**−1.512**	0.848	0.227	0.095
Mosrata^b^	Libya	Eastern Mediterranean	Afro-Sicilian Basin	32°22′N 15°05′E	12	8	28	0.894 ± 0.078	0.0150 ± 0.0044	8.833	− 0.212	0.420	0.140	0.094
Venice Lagoon^b^	Italy	Eastern Mediterranean	Adriatic Sea	45°27′N 12°16′E	11	7	10	0.909 ± 0.066	0.0060 ± 0.0009	3.563	0.186	−1.205	0.149	0.064
Pomer^a^	Croatia	Eastern Mediterranean	Adriatic Sea	44°49′N 13°53′E	20	15	19	0.953 ± 0.035	0.0047 ± 0.0008	2.810	−**1.797**	**−11.055**	**0.052**	0.046
Amvrakikos^a^	Greece	Eastern Mediterranean	Ionian Sea	39°1.2’N20°45.3′E	13	7	11	0.731 ± 0.133	0.0028 ± 0.0008	1.692	**−2.112**	**−2.833**	**0.093**	0.044
Lefkada	Greece	Eastern Mediterranean	Ionian Sea	38°49.6’N 20°43′E	14	8	27	0.824 ± 0.098	0.0088 ± 0.0045	5.208	**−1.648**	−0.315	0.184	0.042
Navarino^a^	Greece	Eastern Mediterranean	Ionian Sea	36°57’N 21°39.7′E	10	7	26	0.867 ± 0.107	0.0220 ± 0.0040	12.911	1.930	1.481	0.230	**0.241**
Alexandroupolis	Greece	Eastern Mediterranean	Aegean Sea	40°51′N 25°52′E	12	7	20	0.773 ± 0.128	0.0083 ± 0.0024	4.893	−1.146	− 0.074	**0.097**	0.053
Izmir Bay	Turkey	Eastern Mediterranean	Aegean Sea	38°27′N 27°05′E	6	3	4	0.600 ± 0.215	0.0022 ± 0.0009	1.333	**−1.295**	0.296	0.235	0.737
Enez Dalyan Lagoon	Turkey	Eastern Mediterranean	Aegean Sea	40°42′N 26°03′E	20	13	23	0.853 ± 0.080	0.0050 ± 0.0014	2.973	**−2.081**	**−6.804**	**0.062**	0.055
Dardanelles Strait	Turkey	Eastern Mediterranean	Sea of Marmara	40°13′N 26°32′E	5	3	12	0.700 ± 0.218	0.0081 ± 0.0036	4.800	**−1.205**	2.225	0.291	0.470
Prince’s Islands	Turkey	Eastern Mediterranean	Sea of Marmara	40°52′N 29°05′E	12	6	16	0.758 ± 0.122	0.0065 ± 0.0022	3.833	−1.193	0.323	0.142	0.084
Bosphorus Strait	Turkey	Eastern Mediterranean	Sea of Marmara	41°12′N 29°06′E	7	6	7	0.952 ± 0.096	0.0050 ± 0.0007	2.952	0.172	−2.275	0.165	0.065
Total					263	106	97	0.912 ± 0.012	0.0211 ± 0.0009	12.389	− 0.650	**−23.885**	0.062	0.023

Significant values are in bold. Non-significant values for the raggedness index (*rg*) accept the null hypothesis of expectation under a sudden demographic expansion model. ^a^: Data examined by Ragionieri and Schubart [35]. ^b^: Data examined by Deli et al. [12]. Examined specimens of the population of Navarino (labelled as Peloponnesus in Ragionieri and Schubart [35]) were those assigned to Cox1 type I

### Cox1 gene amplification and sequencing

The decapod primers COL6a and COH6 (specifically designed for decapod crustaceans; see [46]) or the universal primers LCO1490 and HCO2198 [47] were used to amplify parts of the mitochondrial Cox1 gene. The adopted PCR mixture and thermocycling conditions were detailed in Deli et al. [12]. After being loaded on a 1.5% agarose gel and visualized under UV light, strong products were outsourced for sequencing with primer COL6a or HCO2198 to LGC Genomics (Berlin) or Macrogen Europe (Netherlands). The obtained sequences were visually inspected with Chromas Lite 2.1.1 [48], aligned with Clustal W as implemented in BioEdit [49], and trimmed to a 587 bp fragment for subsequent analyses. Sequences of Cox1 haplotypes, corresponding to the retrieved three haplogroups, were submitted to GenBank (accession numbers: MG798798-MG798903).

### Statistical analyses

#### Intra-population genetic diversity and detection of selection signatures

We assessed the nucleotide composition of the analysed Cox1 fragment with MEGA version 7.0.18 [50]. In order to assess genetic diversity for each population as well as for the total dataset, we computed the number of haplotypes (*N*h), number of polymorphic sites (*N*ps), haplotype diversity (*h*; [51]), nucleotide diversity (*π*; [51, 52]), and mean number of nucleotide differences (K) using DnaSP version 5.10 [53].

To test in how far natural selection is operating on the *C. aestuarii* Cox1 gene, we used the codon-based Z-test of selection for analysis, averaging over all sequence pairs, as implemented in MEGA version 7.0.18 [50]. Codon-based models of molecular evolution are able to infer signatures of selection from alignments of homologous sequences by estimating the relative rates of synonymous (*d*_S_) and nonsynonymous substitutions (*d*_N_) [54]. The relative abundance of synonymous and nonsynonymous substitutions within the examined gene sequences were computed, averaged, and compared for each population as well as for the total dataset. The rejection of the null hypothesis of strict neutrality (H_0_: *d*_N_ = *d*_S_) depends on the tested alternative hypothesis (H_A_): (a) lack of neutral evolution (*d*_N_ is different from *d*_S:_ *d*_N_ ≠ *d*_S_), (b) positive selection (*d*_N_ > *d*_S_) or (c) purifying selection (*d*_N_ < *d*_S_). A two-tailed test was implemented to reject neutral evolution, while one-tailed tests were used to check for positive and purifying selection, respectively. The variance of the difference between synonymous and nonsynonymous substitutions was computed using the bootstrap method (1000 pseudoreplicates). Analyses were conducted using the Nei and Gojobori [55] procedure.

#### Intraspecific evolutionary relationships among Cox1 haplotypes

A statistical parsimony network, constructed with the software TCS version 1.21 [56] under the 95% probability criterion for a parsimonious connection [57, 58], allowed inferring intraspecific evolutionary relationships among the Cox1 haplotypes of *C. aestuarii*. Based on the outcome of the TCS analysis, we also examined the distribution pattern of the discerned divergent haplotypes (or Cox1 haplogroups) across the surveyed geographic region.

#### Relationship between phylogeny and the geographical distribution of haplotypes

Based on the particular pattern of evolutionary relationships among Cox1 haplotypes (yielding various differentiated haplogroups that were restricted to specific geographic regions), we assessed the relationship between phylogeny and the geographical distribution of the recorded haplotypes by measuring levels of population subdivision, using both unordered (*G*_ST_) and ordered haplotypes (*N*_ST_). Estimation and comparison of these parameters was based on the methods described by Pons and Petit [59, 60] using PERMUT & CPSRR version 2.0 [60]. If *N*_ST_ is significantly higher than *G*_ST_, it usually indicates the presence of phylogeographic structure [60, 61].

#### Divergence estimation among resulting mitochondrial Cox1 haplogroups

In order to elucidate the evolutionary history of *C. aestuarii* across the surveyed geographic region, we estimated divergence time between the discerned mitochondrial haplogroups. For this purpose, we applied different models and calibration strategies in order to obtain a comprehensive estimate, minimizing uncertainties due to different model assumptions. First, we applied a known biogeographical event calibration using interspecific sequences (considering the speciation event between Mediterranean and Atlantic green crab species). Subsequently, the entire intraspecific dataset (all examined sequences of *C. aestuarii* in this study) was used for the divergence estimate, applying a specifically determined clock rate of the examined genetic marker for the genus *Carcinus* [23].

In a first analysis, we considered the closure of the Strait of Gibraltar at the beginning of the Messinian Salinity Crisis (5.59 million years ago; [8]) as calibration point for divergence estimation. Indeed, the completely interrupted contact between the Mediterranean Sea and the Atlantic Ocean, during the Messinian Salinity Crisis, is thought to provide the responsible geographic barrier for the speciation of the Mediterranean green crab *C. aestuarii* and its Atlantic sister species *C. maenas* (see [26, 62]). Divergence estimations were carried out in BEAST version 1.7.5 [63]. Prior to the analysis, the most appropriate model of sequence evolution for the dataset was selected using MODELTEST version 3.7 [64] based on Akaike Information Criterion scores. We included only unique haplotype sequences, corresponding to the encountered *C. aestuarii* haplogroups and the Atlantic sister species *C. maenas* respectively. Notably, for such kind of analysis, the simplest tree priors are the one parameter Yule model [65] and the two-parameter Birth-Death model [66, 67]. We used the latter model, since it has been suggested as an appropriate null model for species diversification [66]. In order to test for the right clock model, analyses were carried out first with a strict clock and repeated with an uncorrelated lognormal relaxed clock [68]. Since the parameter of the standard deviation of the uncorrelated lognormal relaxed clock was significantly different from zero (ucld.stdev = 0.28, 95% HPD: 1.13 10^− 4^ -0.54), highlighting variation in rates among branches, final analyses were run enforcing a relaxed molecular clock model. Uncertainty on the divergence time was modelled using a normal prior with a standard deviation of 55,000 years [23]. The normal distribution is considered a useful calibration prior when applying a biogeographical date.

In order to check the consistency of dating results, additional analyses were performed involving only the examined intraspecific data of *C. aestuarii* from this study and implementing the coalescent tree prior that is typically used when all the samples are from the same species [69]. Both strict molecular clock and lognormal relaxed molecular clock models were compared using Bayes factors (BF) to test which of these two clock models best fitted our intraspecific data. We used TRACER version 1.5 [70] to compare twice the difference in the marginal model posterior likelihoods (MMPLs) as estimated from the harmonic mean of the sample of posterior trees for each scenario. Values of 2ln (BF) > 10 were considered as very strong evidence for a given model to be more likely than another [71]. As the Bayes factors indicated a much better fit for the strict clock model (MMPL = − 9484.4) than for a relaxed clock model (MMPL = − 9984.3) (2 ln(BF) =12.428), the final analyses were carried out with a strict molecular clock, and assuming the generalised time reversible (GTR) model of sequence evolution [72], as calculated by MODELTEST version 3.7 [64]. The specifically estimated mutation rate for *Carcinus* of 3.86% per Myr (see [23]) was used to calibrate the genealogy and date tMRCA of Cox1 lineages. This complementary strategy was implemented to minimise errors of divergence times estimation inferred from the use of deep calibration points [73].

For all kinds of Bayesian analyses, the Markov chain Monte Carlo (MCMC) simulations were run for 100 million steps and sampled every 1000 steps. The corresponding outputs were reviewed in TRACER version 1.5 [70] for robustness, and the resultant trees were summarized in TreeAnnotator (implemented in BEAST). The final results are presented with FigTree version 1.4.0 [74].

#### Population genetic structure and phylogeographic examination

Overall population genetic differentiation (assessed by one-level Analysis of molecular variance [75]) as well as detailed pairwise comparison of genetic differentiation were estimated in ARLEQUIN version 3.1 [76], using the two fixation indices: *Φ*_ST_ (implementing the Tajima-Nei model, appropriate for unequal nucleotide frequencies [77]) and *F*_ST_ (based on haplotypic frequency). For both kinds of analyses, the resulting significant values were calculated from 10,000 permutations. B-Y FDR correction [78] was then applied to yield the exact level of significance (critical value = 0.00830 with 231 hypothesis tests and alpha = 0.05). In order to characterize patterns of genetic structure, i.e., identify differentiated genetic groups of populations, we performed a non-metric Multidimensional Scaling (MDS), through PAST version 2.17 [79], based on Tajima-Nei genetic distances. In order to test the hypothesis that patterns of genetic differentiation are caused by isolation by distance (IBD), we ran the Mantel test [80] for pairwise matrices between geographical and genetic distances. The Mantel test was performed with the software AIS (Alleles in Space) version 1.0 [81]. The statistical significance of the test was assessed by running 10,000 random permutations. Population genetic structure of *C. aestuarii* was also examined (by means of two-level AMOVAs) under various biogeographic hypotheses, testing the significance of population structure among Mediterranean basins (Western Mediterranean vs. Eastern Mediterranean), or among defined regions within basins (Algerian Basin vs. Afro-Sicilian Basin vs. Adriatic Sea vs. Ionian Sea vs. Aegean Sea vs. Sea of Marmara). Partitioning of *C. aestuarii* genetic variation was also assessed based on the outcome of haplotype network (among the main groups of populations defining each haplogroup) and pairwise comparisons of genetic differentiation, as well as the MDS plot.

The spatial analysis of molecular variance (SAMOVA) approach, implemented in SAMOVA version 1.0 [82], without a prior population structure definition, was also used to infer the likely number of hierarchical groups explaining most of the retrieved genetic structure within *C. aestuarii*. The software was run with 100 random initial conditions for 10,000 iterations, with number of tested groups (*K*) ranging from 2 to 8.

#### Demographic history

Three neutrality tests (Tajima’s *D* [83], Fu’s *F*s [84], and Ramos-Onsins and Rozas’s *R*_2_ [85]) were used to assess deviation from neutrality, and examine the demographic history of the Mediterranean green crab. Both *D* and *F*s indices were estimated in ARLEQUIN, while *R*_2_ statistic was calculated in DnaSP. The examination of deviation from neutrality by all three indices was based on 1000 coalescent simulations. A scenario of population expansion is likely supported by significantly negative *D* and *F*s values as well as significant *R*_2_ (in small population sizes). The Harpending’s raggedness index *rg* [86] was also used to examine demographic changes in *C. aestuarii* according to the population expansion model implemented in ARLEQUIN. A total of 10,000 replicates allowed testing the significance of the *rg* index. The four above mentioned parameters (*D*, *F*s, *R*_2_, and *rg*) were applied to each examined population, the overall dataset, as well as the genetically differentiated geographic groups (as inferred mainly by SAMOVA).

Since deviations from neutrality are usually caused by changes in effective population size, we also applied Bayesian Skyline plots (BSP) [87] to explore the magnitude of historical demographic events. Notably, this Bayesian approach could allow the inference of detailed and realistic population size function [88], and also yield accurate estimation of expansion events [89]. BSP plots were generated for the geographic groups retrieved by SAMOVA. Analyses were carried out in BEAST version 1.7.5 considering a GTR model (as already calculated by MODELTEST version 3.7) and a strict molecular clock (confirmed as the best model fitting the examined intraspecific data when compared with lognormal relaxed molecular clock using Bayes factors (BF) method). The specific mutation rate of 3.86% per Myr, as estimated for *Carcinus* by Marino et al. [23], was implemented in the analysis in order to date expansion event. Pattern of effective population size evolution through time was assessed taking into account a generation time of approximately two years in the green crab [90]. Two independent MCMC (each with 50,000,000 iterations) were carried out. Following the removal of the first 10% iterations (5,000,000) as burn-in, the remaining replicates were combined in LogCombiner [63] and summarized as BSPs after checking their convergence (Effective Sample Sizes (ESS) of all parameters > 200 for each group) in TRACER version 1.5.

## Results

### Genetic diversity and detection of selection signatures

**Fig. 2:**
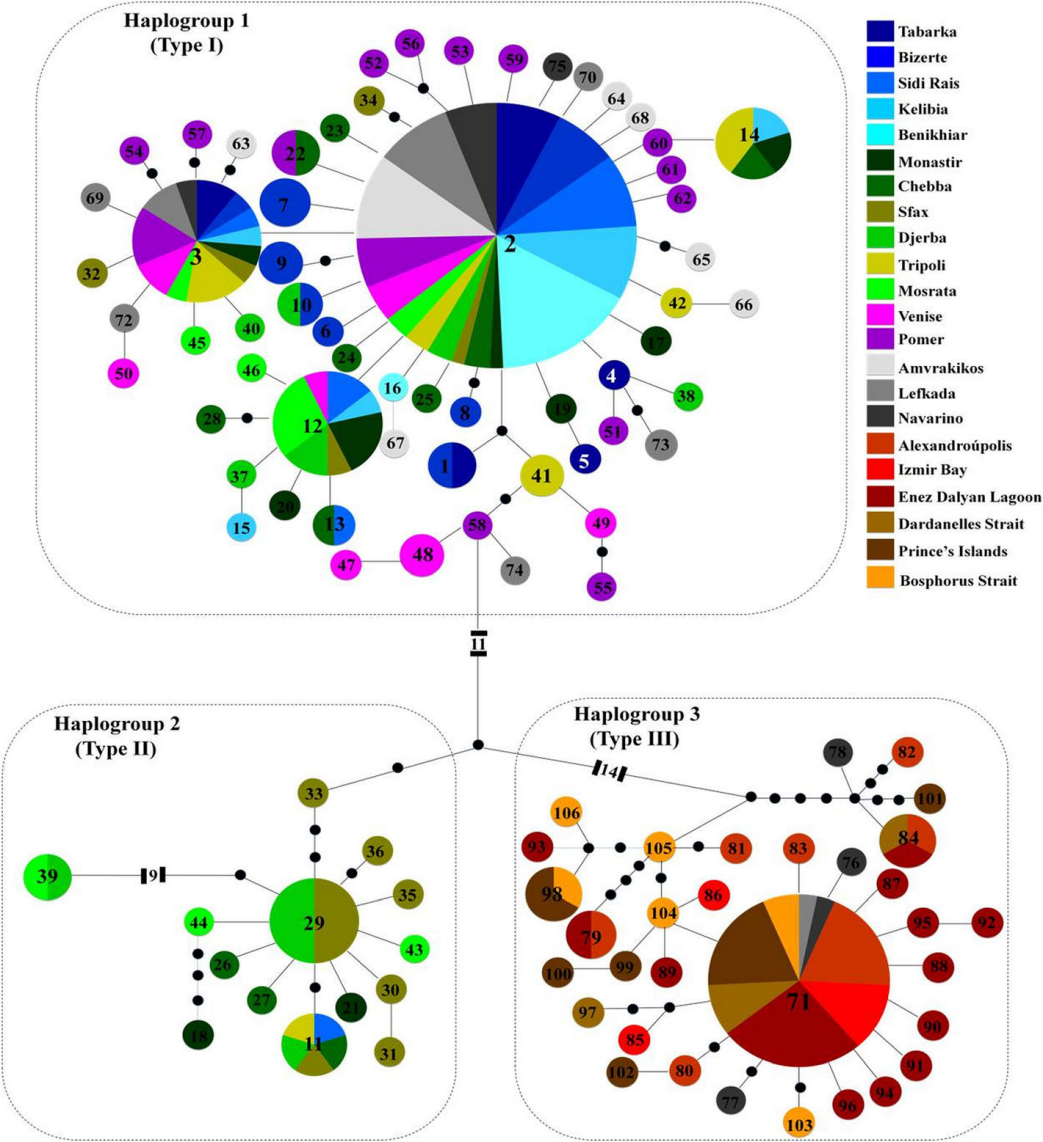
TCS haplotype network of *Carcinus aestuarii*, based on the alignment of 587 bp of the mitochondrial gene Cox1, showing the relationships among the recorded haplotypes. Haplotype 2 corresponds to the ancestral haplotype. Small black circles correspond to missing (or hypothetical) haplotypes. Each line between two points represents one mutational step. Circle sizes depict proportions of haplotypes; the smallest corresponds to 1 and the largest to 67 individuals

Sequences corresponding to the mtDNA Cox1 gene from 263 individuals and 22 locations of *C. aestuarii* were included in the analyses. Of these, 76 were newly obtained, proofread and aligned. The resulting alignment had to be trimmed to a length of 587 basepairs. A total of 97 nucleotide sites were variable, of which 58 were parsimony-informative. Nearly 40% of the examined sequences were unique and allowed the identification of 106 haplotypes (Fig. 2 and Table 1). The nucleotide composition of the analyzed fragment showed an A-T bias (C = 18.81%; T = 36.12%; A = 26.62%; G = 18.45%), which is typical for invertebrate mitochondrial DNA [91].

Genetic diversity analyses of this mitochondrial dataset revealed high total haplotype (*h* = 0.912 ± 0.012) and nucleotide (*π* = 0.0211 ± 0.0009) diversities (Table 1). A high level of the mean number of nucleotide differences (K) was also inferred (K = 12.389). The values of this parameter ranged from 0.166 in the population of Benikhiar to 12.911 in Navarino (Table 1).

The codon-based Z-test of selection allowed the rejection of the null hypothesis of strict-neutrality (*d*_N_ = *d*_S_) for all examined populations (except Benikhiar), as well as for the total dataset (Table 2). Notably, the null hypothesis of strict-neutrality was rejected because of purifying selection (*d*_S_-*d*_N_ = 6.256, *P* = 0.000), as no significant positive selection was detected for all investigated populations of *C. aestuarii* as well as for the whole dataset (*d*_N_-*d*_S_ = − 6.013, *P* = 1.000) (Table 2).

**Table 2:** Codon-based Z-test of selection for analysis averaging over all sequence pairs. Alternative hypotheses of neutrality, positive selection and purifying selection were tested for each population as well as for the total dataset of *Carcinus aestuarii*. For each population, examined specimens were assigned to the corresponding Cox1 type

Population	Number of specimens	Cox1 Type			Lack of neutral evolution (*d*_N_=/=*d*_S_)		Positive selection (*d*_N_ > *d*_S_)		Purifying selection (*d*_N_ < *d*_S_)	
		I	II	III	Test statistic (*d*_N_-*d*_S_)	*P* value	Test statistic (*d*_N_-*d*_S_)	*P* value	Test statistic (*d*_S_-*d*_N_)	*P* value
Tabarka	10	10	–	–	−2.381	0.019	−2.509	1.000	2.403	0.009
Bizerte	15	15	–	–	−2.409	0.018	−2.371	1.000	2.386	0.009
Sidi Rais	11	10	1	–	−4.545	0.000	−4.538	1.000	4.631	0.000
Kelibia	10	10	–	–	−2.417	0.017	−2.549	1.000	2.478	0.007
Benikhiar	12	12	–	–	−0.979	0.330	−1.007	1.000	1.010	0.157
Monastir	11	9	2	–	−5.284	0.000	−5.271	1.000	5.274	0.000
Chebba	12	9	3	–	−5.580	0.000	−5.671	1.000	5.645	0.000
Sfax	15	5	10	–	−5.035	0.000	−5.045	1.000	5.090	0.000
Djerba	14	8	6	–	−5.121	0.000	−5.145	1.000	5.172	0.000
Tripoli	11	10	1	–	−4.672	0.000	−4.755	1.000	4.818	0.000
Mosrata	12	9	3	–	− 5.216	0.000	− 5.169	1.000	5.246	0.000
Venice Lagoon	11	11	–	–	−3.126	0.002	−3.105	1.000	3.317	0.001
Pomer	20	20	–	–	−4.108	0.000	−4.064	1.000	4.213	0.000
Amvrakikos	13	13	–	–	−3.480	0.001	−3.554	1.000	3.514	0.000
Lefkada	14	13	–	1	−5.091	0.000	−5.108	1.000	4.886	0.000
Navarino	10	6	–	4	−5.234	0.000	−5.097	1.000	4.919	0.000
Alexandroupolis	12	–	–	12	−4.282	0.000	−4.209	1.000	4.190	0.000
Izmir Bay	6	–	–	6	−2.023	0.045	−2.089	1.000	2.084	0.020
Enez Dalyan Lagoon	20	–	–	20	−4.541	0.000	− 4.577	1.000	4.540	0.000
Dardanelles Strait	5	–	–	5	−3.547	0.001	−3.566	1.000	3.665	0.000
Prince’s Islands	12	–	–	12	−3.766	0.000	−3.659	1.000	3.739	0.000
Bosphorus Strait	7	–	–	7	−2.649	0.009	−2.599	1.000	2.626	0.005
Total	263	170	26	67	−6.224	0.000	−6.013	1.000	6.256	0.000

For each test hypothesis, the probability of rejecting the null hypothesis of strict-neutrality (*d*_N_ = *d*_S_) as well as the probability of rejecting the null hypothesis of strict-neutrality (*d*_N_ = *d*_S_) in favor of the alternative hypothesis of positive selection (*d*_N_ > *d*_S_) or purifying selection (*d*_N_ < *d*_S_) were expressed as *P* values. Values of *P* less than 0.05 are considered significant. *d*_S_ and *d*_N_ are the numbers of synonymous and nonsynonymous substitutions per site, respectively

### Intraspecific evolutionary relationships among Cox1 haplotypes

The phylogeographic relationships among the 106 recorded haplotypes, as inferred by the TCS statistical parsimony procedure, revealed a remarkable divergence among three haplogroups. These haplogroups will from now on be referred to as types I, II (see also Deli et al. [12]), and III. They are all characterized by a star-like shape centred around three main haplotypes: 2, 29 and 71 respectively (Fig. 2). Type III was found to be the most divergent, separated by at least 28 mutations from type I, and 17 mutations from type II. The most frequent haplotype 2 was found in 67 individuals and in all populations except those from northern Greece (Alexandroupolis) and Turkey (Izmir Bay, Enez Dalyan Lagoon, Dardanelles Strait, Prince’s Islands and Bosphorus Strait) (Additional file: Table S1). Haplotype 2 was distinguished from haplotypes 29 and 71 by 21 and 35 mutational steps, corresponding to sequence divergence rates of 3.40% and 5.96%, respectively. The main haplotypes 29 and 71, representative of types II and III respectively, were separated by 24 mutational steps, corresponding to a rate of sequence divergence of 4.08%.

### Relationship between phylogeny and the geographical distribution of haplotypes

**Fig. 3:**
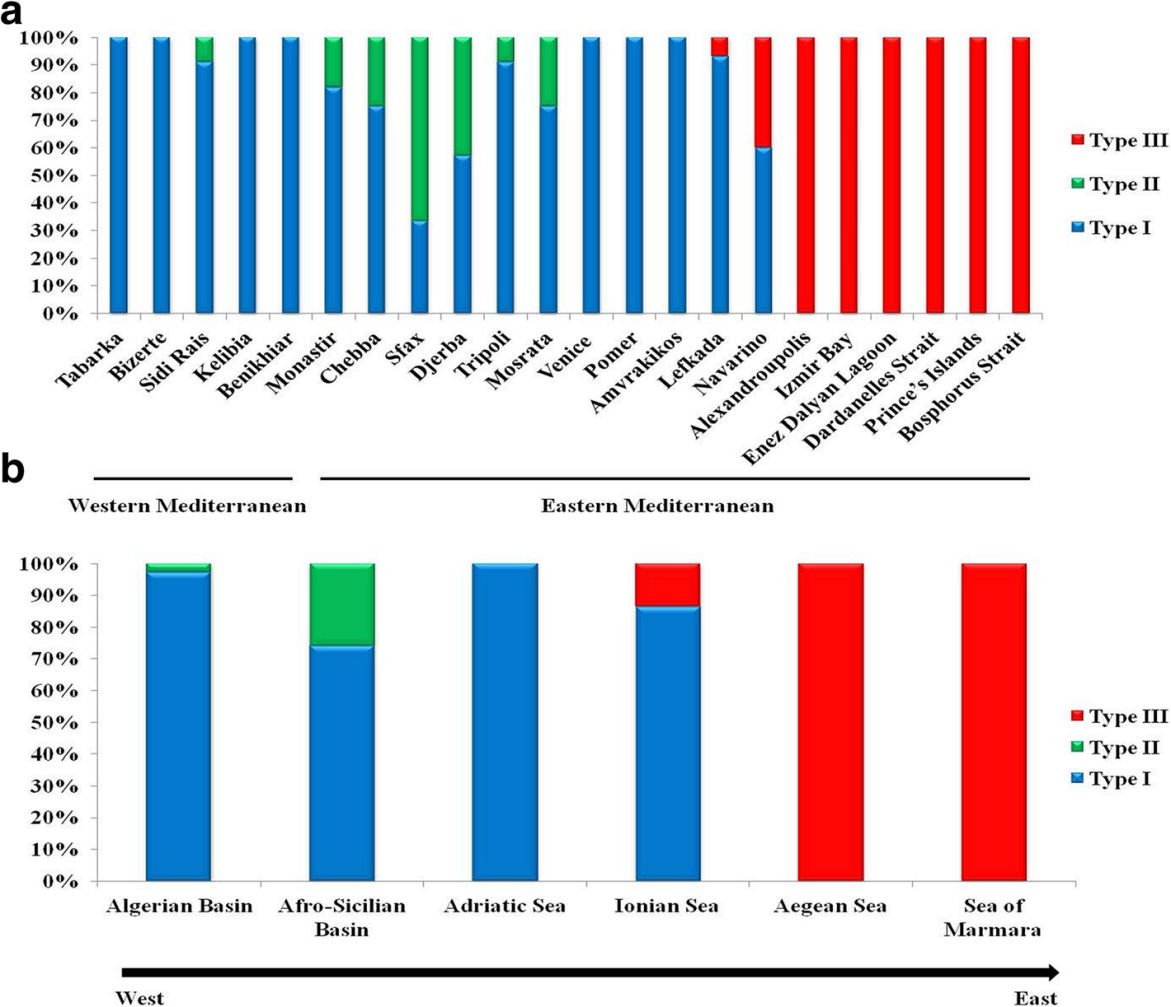
Distribution patterns of types I, II and III of *Carcinus aestuarii* Cox1 gene across western and eastern Mediterranean basins (**a**) as well as geographic regions (**b**) (as defined in Table 1)

While type I sequences proved to be present in almost all examined populations, the genetic distinctiveness of the other two haplogroups hints at the existence of a remarkable regional geographic structure within *C. aestuarii* across the Eastern Mediterranean Basin. Indeed, type II sequences were only found in Tunisian (Monastir, Chebba, Sfax and Djerba) and Libyan (Tripoli and Mosrata) populations. On the other hand, type III sequences were mostly found in specimens from Greece (with increasing frequency in Lefkada, Navarino, and Alexandroupolis) and Turkey (Figs. 2 and 3a) to the extent that Alexandroupolis and the Turkish populations do not contain Cox1 type I, present in all other examined populations (Fig. 3a). Hence, the exclusive pattern of distribution of Cox1 type III in the Aegean Sea and the Sea of Marmara (Figs. 2 and 3b) and its lacking in the remaining investigated distribution range supports the existence of a phylogeographic break across the Eastern Mediterranean. The likely existence of geographic separation was supported and confirmed by the outcome of PERMUT analyses. Calculations of *N*_ST_ (0.211) and *G*_ST_ (0.121) revealed that the *N*_ST_ value is significantly higher than the *G*_ST_ value (*P* < 0.05), inferring significant relationship between phylogeny and the geographical distribution of haplotypes, and indicating the existence of marked phylogeographic structure within the examined material.

### Distribution patterns of the divergent Cox1 types across the surveyed geographic region and clinal variation analysis

All three recorded Cox1 types occur in the Eastern Mediterranean (Fig. 3a). This clearly contrasts with distribution patterns in the Western Mediterranean, where almost exclusively one genetic type (type I) prevails (as evidenced by the present study (Fig. 3a) and the former study by Ragionieri et al. [35]). It should be noted that no population harbours all three types. Both genetic types I and II have been shown to co-exist only in the Tunisian and Libyan populations; while types I and III are recorded together in the two Greek populations of Lefkada and Navarino (Fig. 3a). Of particular interest to this study is the pattern of geographic distribution and transition of Cox1 types I and III along the Greek coastline, which might reflect a marked longitudinal cline. The proportion of type I, for example, decreased notably from the westernmost population of Amvrakikos (exclusively present) to the easternmost location of Alexandroupolis (where it was lacking) (Fig. 3a). This can also be clearly observed at the regional level. Indeed, a gradual decrease in proportion of type I was shown from the Adriatic Sea to the Aegean Sea (Fig. 3b).

### Divergence estimation among Cox1 haplogroups

The outcome of Bayesian phylogenetic analysis, as implemented in BEAST, confirmed the results inferred from the TCS parsimony procedure, yielding a noticeable separation between the highly differentiated type III and the monophyletic group composed of both types I and II. Assuming i) a 5.59 Myr split (see [8]) between *C. aestuarii* and *C. maenas* as calibration point and ii) a Birth-Death prior and uncorrelated lognormal relaxed clock, divergence among type III and the monophyletic group composed of types I and II was estimated to occur approximately around 1.54 Mya (95% HPD - high posterior density interval: 0.88–2.67 Mya). Using a strict molecular clock with a species-specific mutation rate of 3.86% per Myr (calculated and used by Marino et al. [23] for *Carcinus*), and assuming a GTR model of sequence evolution and a coalescent tree prior, involving all the intraspecific data of *C. aestuarii*, the estimated time of the split between Cox1 type III and both types I and II was relatively younger (0.8 Mya; 95% HPD: 0.54–1.05 Mya).

### Population genetic structure and phylogeographic examination

**Fig. 4:**
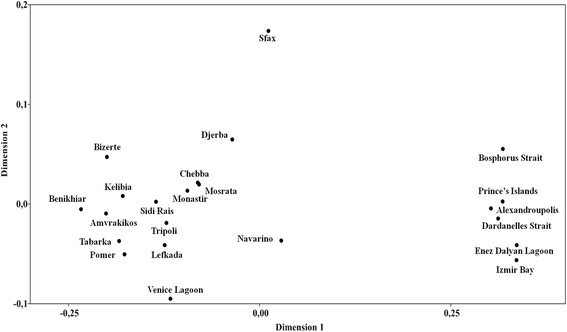
Multidimensional scaling plot based on *Φ*_ST_ (Tajima-Nei distances) values between examined populations of *Carcinus aestuarii*

The one-level AMOVA gives evidence for strong and highly significant genetic differentiation among the examined populations of *C. aestuarii* based on Tajima-Nei distances (*Φ*_ST_ = 0.615, *P* < 0.001) and haplotype frequencies (*F*_ST_ = 0.109, *P* < 0.001). This differentiation was more pronounced based on nucleotide divergence (more than 61% of the variation among populations). Pairwise comparisons of genetic differentiation, estimated from nucleotide divergence and haplotype frequencies, also yielded significant differences for most comparisons and revealed, in particular, a clear genetic distinctiveness of the populations of Alexandroupolis, Izmir Bay, Enez Dalyan Lagoon, Dardanelles Strait, Prince’s Islands and Bosphorus Strait, after B-Y FDR correction (Table 3). The outcome of pairwise comparisons between these latter populations did not reveal significant differences, highlighting the existence of pronounced genetic divergence between populations from the Aegean Sea and the Sea of Marmara, and those assigned to the remaining distribution area. This trend of separation was also confirmed by the outcome of a Multidimensional Scaling (MDS) analysis, based on Tajima-Nei distances (Fig. 4), suggesting a kind of parapatric genetic divergence among two adjacent geographic groups of *C. aestuarii*. A significant relationship was found between genetic and geographic distances (*r* = 0.189, *P* = 0.002) by means of a Mantel Test, supporting an isolation by distance hypothesis to better explain the population separation.

**Table 3:** Pairwise comparisons of genetic differentiation estimated from nucleotide divergence (*Φ*_ST_, below the diagonal) and haplotype frequency (*F*_ST_, above the diagonal). Asterisks indicate significant values (*P* < 0.05) calculated from 10.000 permutations. These significance values were subjected to a B-Y FDR correction [78], rendering a critical value of *P* < 0.0083; values that remain significant after the corection are shown in boldface

	Algerian Basin			Afro-Sicilian Basin								Adriatic Sea		Ionian Sea			Aegean Sea			Sea of Marmara		
	TAB	BIZ	SIR	KEL	BKH	MON	CHE	SFX	DJR	TRP	MOS	VEN	POM	AMV	LFK	NVR	ALX	IZM	EDL	DRD	PRI	BS
TAB	*	0.000	−0.032	−0.045	0.165*	0.090*	0.048	0.103*	0.098*	0.035	0.081	−0.006	0.012	−0.016	−0.044	−0.039	0.235*	0.311*	0.190*	0.267*	0.243*	0.152*
BIZ	0.005	*	0.027	0.028	0.235*	0.061*	0.021	0.070*	0.061*	0.046	0.062*	0.010	0.013	0.025	0.002	−0.007	0.178*	0.240*	0.140*	0.198*	0.185*	0.095*
SIR	0.006	0.034	*	−0.065	0.131*	0.073	0.051	0.115*	0.085*	0.078	0.045	0.011	0.045	−0.019	−0.019	− 0.018	0.258*	0.336*	0.212*	0.294*	0.266*	0.180*
KEL	0.003	0.029	−0.025	*	0.087	0.102*	0.069	0.141*	0.116*	0.077	0.087	0.024	0.052	−0.033	−0.025	−0.022	0.278*	0.361*	0.229*	0.319*	0.286*	0.201*
BKH	0.043*	0.029	0.035*	0.044*	*	0.403*	0.320*	0.385*	0.362*	0.375*	0.374*	0.292*	0.268*	0.107	0.170*	0.203*	0.530*	0.677*	0.443*	0.660*	0.537*	0.511*
MON	0.065	0.100*	−0.048	0.040	0.112*	*	0.012	0.026	0.020	0.025	−0.037	0.007	0.019	0.121*	0.068*	0.050	0.142*	0.204*	0.104*	0.156*	0.149*	0.051
CHE	0.103	0.136*	−0.015	0.092	0.139	−0.063	*	0.020	0.022	0.009	0.033	0.007	−0.005	0.058	0.027	0.006	0.121*	0.180*	0.084*	0.133*	0.128*	0.030
SFX	0.512*	0.546*	0.396*	0.511*	0.557*	0.259*	0.187*	*	−0.030	0.047	0.043	0.038	0.029	0.129*	0.081*	0.062*	0.139*	0.198*	0.103*	0.153*	0.146*	0.052*
DJR	0.254*	0.293*	0.123	0.246*	0.300*	0.019	−0.019	0.043	*	0.067*	0.021	0.039	0.039*	0.109*	0.075*	0.055	0.155*	0.216*	0.118*	0.172*	0.163*	0.069*
TRP	0.008	0.056*	−0.039	0.013	0.072*	−0.027	− 0.009	0.369*	0.119	*	0.057	0.000	−0.000	0.103*	0.029	0.023	0.169*	0.234*	0.129*	0.187*	0.176*	0.080*
MOS	0.107	0.143*	−0.020	0.085	0.154*	−0.070	− 0.059	0.219*	− 0.015	−0.002	*	0.008	0.031	0.108*	0.063	0.048	0.166*	0.230*	0.128*	0.184*	0.174*	0.079*
VEN	0.107	0.160*	0.047	0.136*	0.219*	0.066	0.086	0.456*	0.206*	0.010	0.076	*	−0.014	0.040	−0.010	−0.017	0.160*	0.224*	0.121*	0.177*	0.167*	0.070*
POM	−0.026	0.027	0.015	−0.000	0.024	0.080	0.119*	0.532*	0.281*	−0.004	0.118*	0.070*	*	0.051*	0.003	−0.007	0.131*	0.187*	0.097*	0.144*	0.138*	0.047*
AMV	−0.008	0.020	0.011	0.002	−0.019	0.091*	0.119	0.532*	0.280*	0.036*	0.134*	0.154*	0.013	*	−0.011	−0.016	0.248*	0.321*	0.204*	0.281*	0.256*	0.171*
LFK	−0.014	0.047*	−0.015	0.016	0.044*	0.014	0.047	0.423*	0.173*	−0.025	0.041	−0.006	0.003	0.026	*	−0.046	0.171*	0.231*	0.136*	0.190*	0.178*	0.100*
NVR	0.262*	0.320*	0.198*	0.270*	0.323*	0.134	0.127	0.297*	0.141*	0.174	0.125	0.218*	0.306*	0.296*	0.138	*	0.138*	0.198*	0.105*	0.153	0.146*	0.066
ALX	0.863*	0.871*	0.807*	0.864*	0.893*	0.731*	0.701*	0.660*	0.643*	0.786*	0.708*	0.816*	0.851*	0.867*	0.771*	0.454*	*	−0.039	−0.027	−0.082	−0.020	0.000
IZM	0.946*	0.936*	0.869*	0.947*	0.978*	0.771*	0.734*	0.687*	0.667*	0.843*	0.743*	0.884*	0.903*	0.938*	0.824*	0.491*	0.001	*	−0.010	−0.080	−0.027	0.035
EDL	0.902*	0.903*	0.861*	0.903*	0.922*	0.802*	0.776*	0.734*	0.724*	0.845*	0.782*	0.867*	0.885*	0.902*	0.829*	0.578*	−0.009	−0.041	*	−0.048	− 0.008	−0.013
DRD	0.898*	0.898*	0.815*	0.899*	0.939*	0.707*	0.670*	0.626*	0.602*	0.785*	0.680*	0.831*	0.868*	0.897*	0.770*	0.385*	−0.107	−0.024	−0.049	*	−0.045	−0.006
PRI	0.889*	0.892*	0.833*	0.890*	0.917*	0.757*	0.727*	0.683*	0.669*	0.812*	0.734*	0.843*	0.870*	0.890*	0.796*	0.493*	−0.030	0.000	−0.017	−0.062	*	−0.019
BS	0.917*	0.913*	0.841*	0.917*	0.951*	0.745*	0.706*	0.657*	0.641*	0.814*	0.716*	0.855*	0.882*	0.912*	0.798*	0.454*	−0.005	0.156	0.054	0.017	−0.029	*

*TAB* Tabarka, *BIZ* Bizerte, *SIR* Sidi Rais, *KEL* Kelibia, *BKH* Benikhiar, *MON* Monastir, *CHE* Chebba, *SFX* Sfax, *DJR* Djerba, *TRP* Tripoli, *MOS* Mosrata, *VEN* Venice Lagoon, *POM* Pomer, *AMV* Amvrakikos, *LFK* Lefkada, *NVR* Navarino, *ALX* Alexandroupolis, *IZM* Izmir Bay, *EDL* Enez Dalyan Lagoon, *DRD* Dardanelles Strait, *PRI* Prince’s Islands, *BS* Bosphorus Strait

Population genetic structure was examined by means of two-level AMOVA, testing for partitioning of genetic variation under alternative biogeographic hypotheses (based on the geographic origin of examined populations, the outcome of haplotype network and the outcome of pairwise comparisons of genetic differentiation). Our results showed significant genetic structure under these various grouping schemes (Table 4). Indeed, besides the significant genetic subdivision across the Siculo-Tunisian Strait (*Φ*_CT_ = 0.164, *P* < 0.05; *F*_CT_ = 0.082, *P* < 0.01), a significant and more pronounced genetic separation within *C. aestuarii* (*Φ*_CT_ = 0.598, *P* < 0.001; *F*_CT_ = 0.079, *P* < 0.001) was also revealed when testing differentiation among Mediterranean sub-basins (biogeographic hypothesis 2; Table 4), defined according to Spalding’s marine ecoregions [92]. Notably, even if all AMOVA results for most of the tested alternative biogeographic hypotheses highlighted significant but relatively similar results, partitioning of the genetic variance among the two adjacent genetic groups (separating the Aegean and Marmara Seas from the remaining distribution area) yielded the highest *Φ*_CT_ and *F*_CT_ levels (*Φ*_CT_ = 0.754, *P* < 0.001; *F*_CT_ = 0.159, *P* < 0.001). It also explained most of the population genetic structure of *C. aestuarii* (more than 75% of genetic variance explained between groups, based on nucleotide divergence; and nearly 16% of inter-group variance discerned according to haplotype frequencies; Table 4). The outcome of spatial analysis of molecular variance (SAMOVA), proposing the number of population groups based on geographical and genetic distances without a prior assumption of group composition, showed that partitioning of variance among groups (*Φ*_CT_) was highest with two hierarchical groups (*K* = 2: *Φ*_CT_ = 0.750, *P* < 0.001; Table 5). It should be highlighted that even if slight differences exist between the generated *Φ*_CT_ values (inferred from the different predefined numbers of group (*K*)), the selected significant grouping of *K* = 2 (corresponding to the highest partitioning of variance among groups) matches perfectly the results inferred from the outcomes of pairwise comparisons of genetic differentiation and MDS plots. Besides, the concordance in the outcome of the two different approaches defining the population structure pattern (with (AMOVA) or without (SAMOVA) a prior structure parameter) consolidate the hypothesis that most of population genetic structure within *C. aestuarii* is explained assuming two hierarchical groups. Accordingly, this perfect agreement between results of different analyses assures the correct delineation of population structure and verifies and confirms the choice of the SAMOVA grouping of *K* = 2. Such pattern of genetic differentiation highlights the existence of a barrier to gene flow between two delineated (and adjacent) geographic groups within *C. aestuarii* in the Eastern Mediterranean Basin. The first group, harbouring mainly types I and II, covered mostly the North African coast as well as the Adriatic and Ionian seas; while the second group, including exclusively type III specimens, characterized the Aegean and Marmara seas.

**Table 4:** Analysis of molecular variance assessing population genetic structure and testing for partitioning of *Carcinus aestuarii* genetic variation under alternative biogeographic hypotheses

Hypothesis tested	Nucleotide divergence (Tajima and Nei distance)	Haplotype frequency
1-Based on the geographic origin of examined populations (separation across the Siculo-Tunisian Strait):		
Western Mediterranean vs. Eastern Mediterranean	*Φ*_SC_ = 0.588*** Φ_ST_ = 0.655*** Φ_CT_ = 0.164*	*F*_SC_ = 0.080*** F_ST_ = 0.156*** F_CT_ = 0.082**
2-Based on the geographic origin of examined populations (separation among the different defined geographic regions):		
Algerian Basin vs. Afro-Sicilian Basin vs. Adriatic Sea vs. Ionian Sea vs. Aegean Sea vs. Sea of Marmara	*Φ*_SC_ = 0.143*** Φ_ST_ = 0.656*** Φ_CT_ = 0.598***	*F*_SC_ = 0.045*** F_ST_ = 0.121*** F_CT_ = 0.079***
3-Based on the outcome of TCS haplotype network:		
(Tabarka + Bizerte + Sidi Rais + Kelibia + Benikhiar + Venice + Pomer + Amvrakikos + Lefkada + Navarino) vs. (Monastir + Chebba + Sfax + Djerba + Tripoli + Mosrata) vs. (Alexandroupolis + Izmir Bay + Enez Dalyan Lagoon + Dardanelles Strait + Prince’s Islands + Bosphorus Strait)	*Φ*_SC_ = 0.087*** Φ_ST_ = 0.701*** Φ_CT_ = 0.673***	*F*_SC_ = 0.018*** F_ST_ = 0.148*** F_CT_ = 0.132***
4-Based on the outcome of genetic differentiation and MDS plot:		
(Tabarka + Bizerte + Sidi Rais + Kelibia + Benikhiar + Monastir + Chebba + Sfax + Djerba + Tripoli + Mosrata + Venice + Pomer + Amvrakikos + Lefkada + Navarino) vs. (Alexandroupolis + Izmir Bay + Enez Dalyan Lagoon + Dardanelles Strait + Prince’s Islands + Bosphorus Strait)	*Φ*_SC_ = 0.167*** Φ_ST_ = 0.795*** Φ_CT_ = 0.754***	*F*_SC_ = 0.045*** F_ST_ = 0.197*** F_CT_ = 0.159***

*: *P* < 0.05; **: *P* < 0.01; ***: *P* < 0.001. The description of populations’ attributions to basins and regions is reported in Table 1. The second grouping scheme corresponds to sub-basins nearly matching marine ecoregions identified by Spalding [92] for the Mediterranean, namely the Adriatic, Ionian and Aegean seas. In particular, we designated the Western Mediterranean marine ecoregion (in Spalding delineation of Mediterranean marine ecoregion) as the Algerian Basin, and re-named the Tunisian Plateau/Gulf of Sidra (as identified in Spalding [92]) as the Afro-Sicilian Basin. We also considered the Black Sea ecoregion (delineated in Spalding [92]) as the Marmara Sea in our grouping scheme

**Table 5:** Results of spatial analysis of molecular variance (SAMOVA), depicting patterns of population structure of *Carcinus aestuarii* for each predefined number of group (*K*)

*K*	Population structure	*Φ*_SC_	*Φ*_ST_	*Φ*_CT_
2	[Tabarka + Bizerte + Sidi Rais + Kelibia + Benikhiar + Monastir + Chebba + Sfax + Djerba + Tripoli + Mosrata + Venice + Pomer + Amvrakikos + Lefkada + Navarino] [Alexandroupolis + Izmir Bay + Enez Dalyan Lagoon + Dardanelles Strait + Prince’s Islands + Bosphorus Strait]	0.165***	0.792***	0.750***
3	[Tabarka + Bizerte + Sidi Rais + Kelibia + Benikhiar + Monastir + Chebba + Djerba + Tripoli + Mosrata + Venice + Pomer + Amvrakikos + Lefkada + Navarino] [Sfax] [Alexandroupolis + Izmir Bay + Enez Dalyan Lagoon + Dardanelles Strait + Prince’s Islands + Bosphorus Strait]	0.081***	0.766***	0.745***
4	[Tabarka + Bizerte + Sidi Rais + Kelibia + Benikhiar + Monastir + Chebba + Djerba + Tripoli + Mosrata + Venice + Pomer + Amvrakikos + Lefkada + Navarino] [Sfax] [Alexandroupolis + Izmir Bay + Enez Dalyan Lagoon + Prince’s Islands + Bosphorus Strait] [Dardanelles Strait]	0.085***	0.762***	0.740***
5	[Tabarka + Bizerte + Sidi Rais + Kelibia + Benikhiar + Monastir + Chebba + Djerba + Tripoli + Mosrata + Venise + Pomer + Amvrakikos + Lefkada + Navarino] [Sfax] [Izmir Bay] [Alexandroupolis + Enez Dalyan Lagoon + Prince’s Islands + Bosphorus Strait] [Dardanelles Strait]	0.089***	0.758***	0.735***
6	[Tabarka + Bizerte + Sidi Rais + Kelibia + Benikhiar + Monastir + Chebba + Djerba + Tripoli + Mosrata + Venice + Pomer + Amvrakikos + Lefkada + Navarino] [Sfax] [Izmir Bay] [Alexandroupolis + Enez Dalyan Lagoon + Prince’s Islands] [Dardanelles Strait] [Bosphorus Strait]	0.093***	0.755***	0.729***
7	[Tabarka + Bizerte + Sidi Rais + Kelibia + Benikhiar + Monastir + Chebba + Djerba + Tripoli + Mosrata + Venice + Pomer + Amvrakikos + Lefkada + Navarino] [Sfax] [Izmir Bay] [Alexandroupolis + Enez Dalyan Lagoon] [Prince’s Islands] [Dardanelles Strait] [Bosphorus Strait]	0.101***	0.750***	0.722***
8	[Tabarka + Bizerte + Sidi Rais + Kelibia + Benikhiar + Monastir + Chebba + Tripoli + Mosrata + Venice + Pomer + Amvrakikos + Lefkada] [Sfax] [Djerba] [Navarino] [Alexandroupolis + Enez Dalyan Lagoon + Prince’s Islands] [Izmir Bay] [Dardanelles Strait] [Bosphorus Strait]	0.015***	0.717***	0.713***

Partitioning of variance among groups (*Φ*_CT_) is highest when there are two hierarchical groups (*K* = 2, shown in bold). *** Significant difference at *P* < 0.001

### Demographic history

Overall, the applied neutrality tests revealed significant deviations from mutation-drift equilibrium for a total of twelve populations (11 by the Tajima’s *D*, 4 by the Fu’s *F*s, and 5 by the Ramos-Onsins and Rozas’s *R*_2_ test; Table 1) suggesting that these populations seem to have experienced a recent expansion event. However, considering the fact that both *D* and *F*s tests have little power to detect departure from neutrality, when sample size is small (less than 15–20 individuals, which is the case for most local populations in the present study), more weight should be put on the outcome of the *R*_2_ test, which is more powerful than either *D* or *F*s tests in case of small sample sizes. Hence, population expansion can be roughly inferred for five populations (Bizerte, Pomer, Amvrakikos, Alexandroupolis, and Enez Dalyan Lagoon); whereas it is uncertain in all the remaining cases. The negative and significant value of Fu’s *F*_*S*_ test, together with the small and non-significant value of Harpending’s raggedness index *rg*, also revealed significant deviations from neutrality for the whole dataset (Table 1), consistent with a scenario of a sudden demographic expansion. Evidence of departure from mutation-drift equilibrium was also recorded for both delineated geographic groups (as identified by SAMOVA). Indeed, all examined neutrality tests resulted in significant values (with marked negative output for both Tajima’s *D* and Fu’s *F*s), associated with small and non-significant values of the Harpending’s raggedness index, for group 1 (*D* = − 1.496, *P* = 0.025; Fu’s *F*_*S*_ = − 24.740, *P* = 0.000; *R*_2_ = 0.041, *P* = 0.035; *rg* = 0.021, *P* = 0.999) and group 2 (*D* = − 1.963, *P* = 0.008; Fu’s *F*_*S*_ = − 20.118, *P* = 0.000; *R*_2_ = 0.038, *P* = 0.001; *rg* = 0.018, *P* = 0.955).

**Fig. 5:**
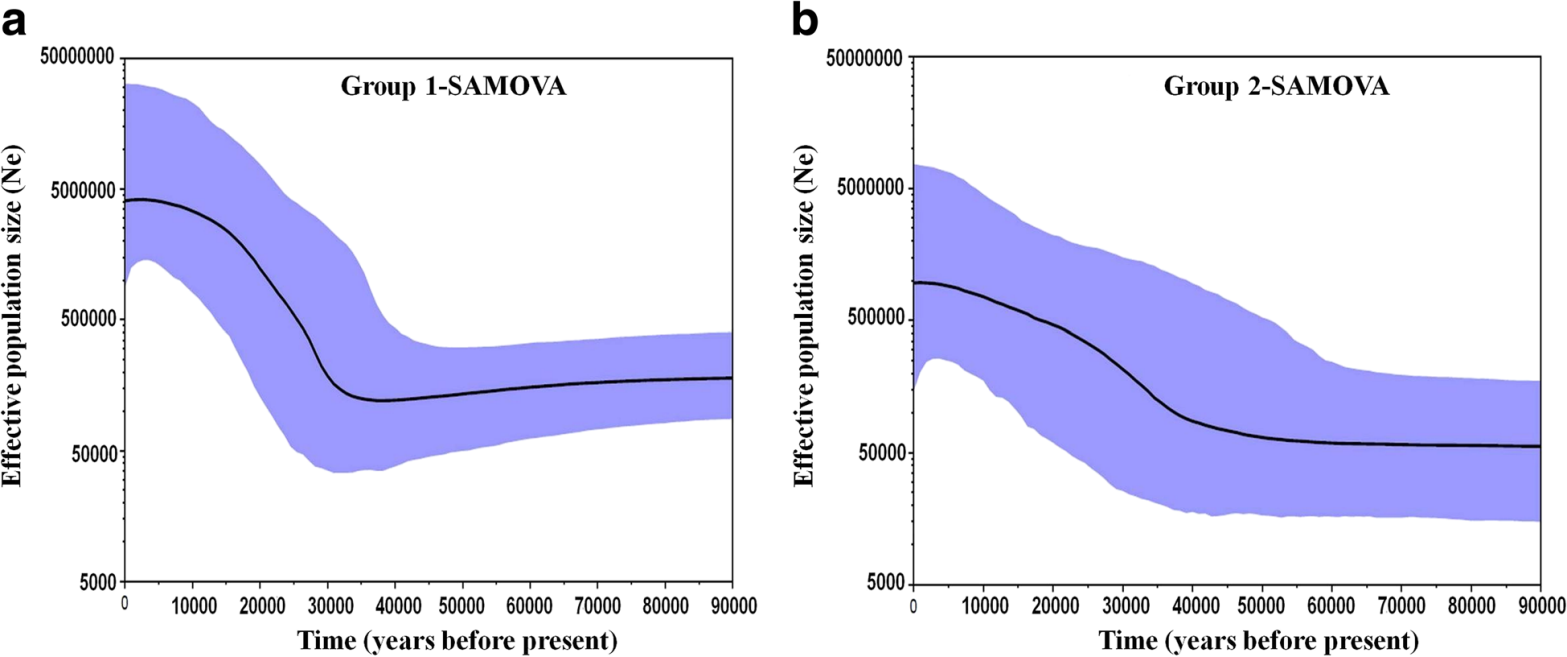
Bayesian skyline plot for the two genetically defined groups of Carcinus aestuarii, as identified by SAMOVA; **a**: group 1; **b**: group 2. Populations, defining both geographic groups, are reported in the results and in Table 5 (K=2). BSP plots showing changes in effective population size (Ne) over time (measured in years before present). The thick solid line depicts the median estimate, and the margins of the blue area represent the highest 95% posterior density intervals

Demographic history of the two geographic groups of *C. aestuarii*, delineated by SAMOVA, was also inferred and detailed from the coalescent approach of the BSP analysis. SAMOVA-group 1 (North African coast + Adriatic and Ionian seas) showed a relatively sudden and recent increase of effective population size over time, following a short phase of size decrease, preceded by quite a stationary period (Fig. 5a). This clearly contrasts with the outcome of BSP plot for SAMOVA-group 2 (Aegean and Marmara seas), yielding remarkably progressive increase in the effective population size (Fig. 5b). Overall, BSP results were well concordant with those inferred from neutrality tests and revealed that the expansion time occurred approximately at about 35,000 years ago (CI: 25,000–42,000 years ago) for SAMOVA-group 1 and about 51,000 years ago (CI: 42,500–69,000 years ago) for SAMOVA-group 2. Notably, the increase of effective size was much more pronounced in the SAMOVA-group 1 (Fig. 5a).

## Discussion

The results of the present study provide new and interesting insights into the genetic composition of *C. aestuarii* across its distribution range, unveiling the existence of a new haplogroup (type III) in the most north-eastern part of the Mediterranean Sea, in addition to two haplogroups (types I and II) previously described by Deli et al. [12]. Type III was previously recorded in four specimens of *C. aestuarii* from the Greek population of Navarino (Peloponnesus) and briefly discussed in Ragionieri and Schubart [35] but the corresponding Cox1 sequences are here analysed for the first time. Overall, concordant results inferred from phylogenetic relationships among the recorded haplogroups, the patterns of their distribution, pairwise comparisons of genetic differentiation (illustrated with MDS plots), and two-levels AMOVA revealed strong genetic divergence among two adjacent groups (type III versus types I-II), separating the Aegean Sea and the Sea of Marmara from the remaining examined populations. Spatial genetic structure, evidenced by SAMOVA, highlighted the existence of a barrier to gene flow between these two delineated geographic groups within *C. aestuarii* and stressing a sharp phylogeographic break across the Eastern Mediterranean.

The existence of these two genetically and geographically differentiated groups confirmed the assumption and previous results that *C. aestuarii* exhibits complex population structure across its distribution range. Earlier investigations by Ragionieri and Schubart [35] and Deli et al. [12] showed marked genetic differentiation between populations from the Eastern and Western Mediterranean basins. In particular, Deli et al. [12] provided evidence for a Pleistocene vicariant event in this species across the Siculo-Tunisian Strait, inferred from marked concordant patterns of mitochondrial and nuclear phylogeographic structure.

The remarkable coexistence of all three Cox1 types (I, II, and III) in the Eastern Mediterranean, being regionally differentiated, allowed us to advance the hypothesis that such particular pattern of mitochondrial diversity within *C. aestuarii* could be the footprint of a complex evolutionary history of the species within the Mediterranean Sea. Type III represents the most distinct haplogroup with high levels of nucleotide divergence. We suggest it to be a genetic isolate that might have survived different episodes of Pleistocene climate changes. During the Quaternary glacial periods, sea level regressions limited the biotic exchange through the Strait of Gibraltar [13] and the Siculo-Tunisian Strait [14]. These historic complete isolation events must have shaped the genetic structure of the marine Mediterranean fauna. In particular, the Mediterranean crustacean fauna has been postulated to originate by repeated or continuous colonization events with adaptation to specialized habitats and adaptive radiation [93]. This could have led to marked genetic differences and a high proportion of endemism in different parts of the Mediterranean Sea [4, 94]. Earlier results by Deli et al. [12] allowed recognition of type II Cox1 haplotypes in the south-western part of the Eastern Mediterranean Basin, postulated to correspond to an eastern Mediterranean endemic isolate, originating from climate oscillations during the Early Pleistocene. Now we can ascertain that this type II seems to be regionally restricted to the African coast of the Eastern Basin.

The particular geographic concentration of *C. aestuarii* with type III mtDNA in the Sea of Marmara and the adjacent Aegean Sea potentially suggests that this isolated haplogroup could have originated in the Black Sea and subsequently dispersed into the Aegean. In this context, we hypothesize that the highly divergent Cox1 type III could be the result of historical isolation mediated by the closure of the Bosphorus Strait during Pleistocene climate shifts [95]. These processes probably caused a total restriction of gene flow and led to genetic divergence between Black Sea populations and their Eastern Mediterranean counterparts. Later on, resumed biotic exchange, following the opening of the Bosphorus Strait 10,000 years ago [95], could have restored the gene flow between the Black Sea and the Eastern Mediterranean, which may partly explain the recorded contemporary genetic structure of the green crab. This advanced scenario for *C. aestuarii* echoes a similar explanation of the discerned phylogeographic pattern in other marine invertebrate from the Black Sea and Aegean Sea, i.e., the bivalve *Mytilus galloprovincialis* (see [96]). Based on the outcome of haplogroup distribution, Kalkan et al. [96] hypothesized that one of the refugial regions of *M. galloprovincialis* could be the Black Sea, with subsequent dispersal of one of the clades (essentially counterparts of type III in the green crab) into the Aegean (via the Black Sea water current), following the onset of connection between both basins. A similar scenario was also proposed for the littoral prawn *Palaemon elegans* genetic type III, with a potential Black Sea origin [11]. Recently, Fratini et al. [97] discerned a specific haplogroup in the marbled crab *Pachygrapsus marmoratus* confined to the Black Sea. The authors stressed on the importance of the biogeographic barrier between the Aegean and Black seas, susceptible of disrupting dispersal and gene flow for many marine species during past and present times and triggering intraspecific diversification in the Mediterranean.

This likely scenario is reinforced by the fact that the Black Sea was repeatedly isolated from the Mediterranean Sea during Pleistocene glaciations and diluted with fresh water during those periods [98]. Svitoch et al. [99] reported that the isolation event resulting in the highest freshwater condition occurred around 18,000–20,000 years ago when the salinity dropped to around 2–4%. Accordingly, we might think that it was impossible for marine invertebrates, i.e., decapods, to survive these conditions. Nevertheless, the Quaternary paleogeography of the Black Sea indicates that it was never an exclusively freshwater basin [100]. Notably, during the Early Pleistocene (around one million years ago) when type III had started evolving, the Black Sea environment was still brackish (during the Pontian and Chaudian epochs), and the green crab could have survived as a ‘Pontic relict’ species [100]. Taking into account these insights, and backing the possibility that *C. aestuarii* could also survive in hypo-saline conditions (given its wide ecological valence [19]), it can therefore make much sense to argue for a potential Black Sea origin of the type III mtDNA in the Mediterranean green crab. Reuschel et al. [11] previously suspected that the genetic type III of *P. elegans* could be a lineage that survived for extended periods in the Black Sea (for example during the Messinian Salinity Crisis). This lineage must be relatively tolerant to brackish waters, considering that it recently invaded the Baltic Sea (characterized by increasingly low salinity from west to east). Similarly, Luttikhuizen et al. [101] argued that other decapod such as the shrimp *Crangon crangon* might also have survived in the Black Sea in brackish waters with salinities of less than 7 ppt.

The marked genetic divergence between both geographically delineated groups (harbouring different Cox1 types) could reflect the impact and likely involvement of historical processes (i.e., Pleistocene climate oscillations; see [4]) in shaping Mediterranean marine diversity. In particular, the significant divergence of type III, being separated by large number of mutational steps from both types I and II, indicates a relatively old separation along the study area and suggests that these mitochondrial clades had been formed and delineated by long-term biogeographic barriers, and their differentiation was probably maintained by restricted historical gene flow. This assumption was confirmed by our dating procedure, based on the use of different models and calibration strategies, placing the divergence between type III and both types I and II at 1.54 Mya to 0.80 Mya. This range of divergence time estimation corresponds approximately to the Early Pleistocene (1.8 to ~ 0.781 Mya according to Riccardi [102]) and coincides with historical episodes of separation between types I and II (1.2 Mya to 0.69 Mya; [12]), providing evidence of simultaneous impact of climate change on genetic structure of the green crab across different parts of the Eastern Mediterranean. It is known that this period was characterized by strong climate shifts and sea-level fluctuations that might have profoundly affected the genetic structure of populations of several Mediterranean marine species (see [4, 103]). Furthermore, the marked gradual transition between type I and type III along the Adriatic, Ionian and Aegean seas may also reflect the impact of historical events in the surveyed region. Genetic clines, such as here observed, could originate from genetic admixture at secondary contact zones, following postglacial recolonization from Pleistocene refugia [104]. Accordingly, we hypothesize that the Ionian Sea could be considered a contact zone between the two defined parapatric divergent groups of *C. aestuarii* within the Eastern Mediterranean, following episodes of historical isolation between the Aegean Sea and the Eastern Mediterranean, as has been reported in several studies [5, 16, 39–45]. Further sampling along the eastern Greek coastline is required in order to test this hypothesis.

The three applied neutrality tests (Tajima’s *D*, Fu’s *F*_*S*_, and Ramos-Onsins and Rozas’s *R*_2_) for both divergent genetic groups of *C. aestuarii* (delineated by SAMOVA) showed significant values, indicating significant deviation from neutrality due to historical factors (such as a population bottleneck or sudden expansion) and contemporary processes (i.e., natural selection). Notably, significant negative *D* and *F*_*S*_ values (interpreted as signatures of historical population expansion, supported by the small and non-significant value of Harpending’s raggedness index *rg*) were retrieved for both geographic groups. Such patterns clearly indicate that these genetic entities have undergone demographic expansion and refer to a loss of equilibrium among mutation, gene flow and genetic drift [83, 84]. Hence, historical processes, rather than contemporary ones, are supposed to be likely involved in triggering the onset of the retrieved phylogeographic pattern. This assumption could be supported by the outcome of Bayesian Skyline Plots analyses. Indeed, different patterns of demographic history were revealed for both groups of *C. aestuarii*, highlighted by more recent expansion event in group 1 (35,000 years ago) than that recorded in group 2 (51,000 years ago). Notably, while both demographic events were found to precede the Last Glacial Maximum (between 26,500–20,000 years ago; [105]), the temporal difference between both genetic groups of *C. aestuarii* points out to the potential intensity and effect of historical processes (i.e., paleoclimate fluctuations) on pattern of genetic diversity evolution. In addition to the observed temporal variation, marked spatial differences in the demographic history of the Mediterranean green crab also have been noticed. While a pattern of sudden population growth was detected for group 1 (composed mainly by types I and II), the species exhibited a pattern of slight and progressive increasing population size in the Aegean Sea and the Sea of Marmara (group 2 harbouring exclusively type III). We may therefore hypothesize that these spatial differences could likely stem from the potential impact of Pleistocene climate fluctuations (alternating glacial-interglacial cycles) on the availability of favourable abiotic conditions (such as suitable temperature and salinity conditions, as well as habitat availability highlighted by suitable ecological niche). In this context, we may attribute the less magnitude of expansion event, recorded in the Aegean and Marmara seas (group 2), to the lack of suitable habitat availability for *C. aestuarii* which could have probably led to population expansion limitation. Indeed, during glacial cycles of the Pleistocene, particularly during the Middle Pleistocene and the last glacial period, the Aegean Sea experienced massive sea-level regression that probably caused half of its area to become land [106, 107]. Moreover, even after the rising of sea level, at the end of the Pleistocene glaciations, the post-glacial recolonization of the Aegean Sea could not have led to considerable increase in the effective population size of *C. aestuarii* owing to the limited areas of the Aegean and Marmara seas.

Alternatively, the recorded pattern of parapatric genetic divergence among the two adjacent groups, with the potential existence of contact zone among both groups in the Ionian Sea, suggest the impact of particular past and present oceanographic patterns (such as marine currents and gyres) across the Eastern Mediterranean. Accordingly, we assume that the recorded pattern of population structure could be mainly caused by the potential effects of the hydrographic isolation of the Adriatic-Ionian and Aegean seas [4, 5] on larval dispersal. In addition to the impact of the strong currents impeding the mixing of the different water bodies at each sub-region, selective forces associated with environmental features in each basin could account for a phylogeographic break. Despite the existence of significant genetic differentiation across the Siculo-Tunisian Strait, as already unveiled by previous investigations [12, 23, 35], the partition maximizing genetic variance among groups (more than 75%) was recorded between the Aegean-Marmara seas and the remaining group of populations (as also confirmed by pairwise comparisons of genetic differentiation, MDS plot and SAMOVA). These results are in concordance with those inferred from other studies on Mediterranean marine invertebrates and vertebrates (see [5, 16, 39–45, 108]), corroborating the isolation of the Aegean Sea from the remaining Mediterranean Sea. In particular, Nikula and Väinölä [39] unravelled genetic subdivision within the bivalve *Cerastoderma glaucum* in the Eastern Mediterranean, highlighting a major phylogeographic break in mitochondrial Cox1 gene sequences between a group of Ponto-Caspian-Aegean Sea haplotypes and those to the west of the Peloponnese peninsula in the Mediterranean. This remarkable pattern of genetic break, within the Eastern Mediterranean, has also been recorded in crustaceans. Notably, Shemesh et al. [108] and later Pannacciulli et al. [18] revealed marked genetic isolation in the barnacle species *Chthamalus montagui* between the western-central Mediterranean Sea and Aegean-Black seas. Gene flow restriction between western and eastern Mediterranean populations was shown to be mainly linked to the hydrographic isolation of the Aegean Sea, and found to be more marked than that associated with the Siculo-Tunisian Strait [45].

Genetic divergence within *C. aestuarii* across the Eastern Mediterranean could also be triggered by historical isolation events resulting in strong bottlenecks. Such isolation events could include Pleistocene hydrographical shifts that allowed repeated isolation and separation of the Aegean Sea from the remaining Eastern Mediterranean [38]. The continuous isolation of both delineated geographic groups (as inferred from SAMOVA) until the present is likely maintained by the Peloponnese anticyclonic front [109–111], which would have prevented gene flow between both geographic groups. What is more, the exclusive existence of type III in the Aegean Sea and Sea of Marmara (with the consequent total lack of types I and II) matches perfectly with the specific oceanographic features of the Sea of Marmara-Aegean Sea area (due to the low salinity surface water mass from the Black Sea flowing from the Black Sea to the Aegean on top of the denser saline Mediterranean waters flowing towards the Black Sea), which should prevent larvae flow from the Mediterranean Sea into the Aegean Sea [39, 45, 112]. Limited genetic connectivity was only revealed between the Ionian and Aegean seas, with occasional dispersal events from the latter area to the former. For instance, type III (predominant in the Aegean Sea) was otherwise only found in the very adjacent locations of Lefkada and Navarino (Ionian Sea) and in lower frequencies, indicating a unidirectional dispersal event.

In addition to the previously discussed impact of palaeoecological history as well as past and present oceanographic processes on shaping the genetic variability and population structure of the green crab *C. aestuarii*, selection on mtDNA haplotypes is another important factor that should be taken into consideration. For instance, the significant genetic patterns, linked to geography within the examined Mediterranean coasts, may strongly suggest that gene flow is not only limited by fluctuating events and neutral processes (as suggested by the observed significant pattern of isolation by distance), but also by environmental factors, i.e., hydrological factors (affecting dispersal) or differential selection (affecting fitness). Reduced dispersal among populations can lead to genetic subdivision of populations and may facilitate local adaptation [113]. The impact of selection on genetic structure has already been suggested in marine invertebrates [114, 115]. Therefore, we may attribute genetic distinctiveness of the eastern group to the specific abiotic features of the Aegean Sea. Water temperatures in the Aegean are known to be influenced by the cold-water masses of low temperature that flow in from the Black Sea through the Dardanelles Straits [116]. The sea surface temperature in the Aegean generally ranges from about 16 to 25 °C. Furthermore, hypersaline Mediterranean water (moving northward along the west coast of Turkey) characterizes the Aegean surface water before being displaced by less dense Black Sea outflow [117]. Hence, we hypothesize that specific environmental features of the Aegean Sea might have exerted selective pressures on the gene pool of *C. aestuarii* in the Eastern Mediterranean. A significant correlation between sea surface temperature and mitochondrial haplotypes has been already recorded in marine species, i.e., the walleye pollock *Theragra chalcogramma* in the North Pacific [118]. Nevertheless, with no detected sign of positive natural selection in examined Cox1 sequences of *C. aestuarii*, this hypothesis remains questionable and would need to be verified.

Overall, the results of the present investigation, along with those already obtained for more western populations of the Mediterranean green crab [12, 35], allow us to postulate the following evolutionary history scenario for *C. aestuarii* throughout its distribution range: During glaciations periods of the Early Pleistocene, dropping sea levels led to the restriction of biotic exchange across the Gibraltar and Siculo-Tunisian straits. The Eastern Mediterranean Basin was more affected by these environmental shifts and experienced desiccation episodes of greater or lesser importance in different parts [119]. Hence, being isolated from the rest of the Mediterranean, an endemic fauna of the Eastern Mediterranean may have originated and evolved a different genetic composition. In this context, climate oscillations during the Pleistocene may have contributed to the simultaneous onset of different genetic isolates (Cox1 Types II and III) in different parts of the Eastern Mediterranean (eastern Mediterranean endemic isolates). Later, the relative impact of historical and contemporary barriers to gene flow, as well as different patterns of postglacial recolonization of *C. aestuarii* from Pleistocene refugia in different parts of the distribution area, might have shaped the current genetic diversity and population genetic structure, such as observed by us. Indeed, in an earlier study by Deli et al. [12], a secondary contact between historically isolated types I and II was noticed in the central Mediterranean (i.e., across the African eastern Mediterranean). The outcome of the present study confirms these earlier insights and revealed further separation between both genetic types (I and II) and the highly diverged type III in the Eastern Mediterranean (see Fig. 1).

## Conclusions

Our study provides new and important insights into the evolutionary history of a highly dispersive benthic decapod crustacean in the Mediterranean Sea. Notably, results of this investigation allow unravelling a sharp phylogeographic break in the Eastern Mediterranean (matching the well-known and reported sharp genetic break between the Aegean Sea and the remaining Mediterranean) and elucidating historical and contemporary processes susceptible of driving such complex phylogeographic structure. Our finding also stress the importance of investigating peripheral areas in the species’ distribution zone to fully understand the distribution of the genetic diversity and unravel hidden genetic units and local patterns of endemism. Lastly, regardless the mechanism involved in shaping pattern of genetic structure of *C. aestuarii*, the two discerned geographic groups deserve to be better investigated. In this sense, analysis of nuclear markers (such as microsatellite loci) in areas where both genetic groups occur is required to confirm this particular divergence pattern. It should be noted that the use of such additional markers may provide changes in phylogeographic patterns and consequent interpretations, as reported by Avise [120], yet still providing complete picture on phylogeographic structure and evolutionary history of the species. In addition, further studies including populations from the Black Sea and those located to the south of the Aegean Sea (i.e. the Levantine Basin) are needed to better understand the evolutionary history of the Mediterranean green crab, and fully characterize and delineate other potential genetic groups or isolates. In addition, further sampling across the Ionian Sea would allow confirming and precisely delineating the geographic occurrence of the observed genetic cline in this study.

## Additional file

Additional file 1: **Table S1.** Geographic distribution of the 106 haplotypes of *C. aestuarii* recorded at the 22 sampling sites within the Mediterranean Sea. (DOCX 44 kb)

## Abbreviations

°C: Degrees celsius
DNA: Deoxyribonucleic acid
mtDNA: Mitochondrial DNA
Mya: Million years ago
Myr: Million years
PCR: Polymerase chain reaction
ppt: Parts per thousand
UV: Ultra violet
